# Procurement and Waste in Hemodialysis: A Detailed Life Cycle Assessment to Reduce Environmental Impact

**DOI:** 10.1016/j.xkme.2026.101517

**Published:** 2026-09-07

**Authors:** James Larkin, Giulia Ligabue, Gaetano Alfano, Rodrigo M. Cadenas, Abass Fehintola, Ingeborg Steinbach, Aycan Yasar, Niccolo Morisi, Marta Arias-Guillen, Marialuisa Caiazzo, Gabriele Donati, Brett Duane

**Affiliations:** 1 School of Dental, Child and Public Health, Trinity College Dublin, Ireland; 2 Department CHIMOMO, University of Modena and Reggio Emilia, Modena, Italy; 3 Nephrology Dialysis and Kidney Transplant Unit, University Hospital Policlinico of Modena, Modena, Italy; 4 Universidad Autonoma de Madrid, Spain; 5 Centre for Sustainable Healthcare, Oxford, United Kingdom; 6 Mozarc Medical, Mirandola, Italy

**Keywords:** Hemodialysis, dialysis, environmental impact, life cycle assessment, sustainable health care, medical waste, resource optimization, kidney failure, kidney replacement therapy, carbon footprint

## Abstract

**Rationale & Objective:**

Hemodialysis (HD) is a resource-intensive treatment associated with high environmental impact. This study aimed to quantify the environmental burden of HD procurement and waste and identify key contributors to emissions, energy use, and water consumption.

**Study Design:**

Life cycle assessment study.

**Setting & Participants:**

Data were collected from the Nephrology Dialysis and Kidney Transplant Unit at the University Hospital Policlinico of Modena, Italy. The analysis reflects 1 patient-year of HD treatment.

**Exposure(s) or Predictor(s):**

Procurement and life cycle of HD consumables and equipment.

**Outcome(s):**

Greenhouse gas emissions (kg CO_2_-equivalent), primary energy demand (MJ), and water use (m^3^).

**Analytical Approach:**

A life cycle assessment was conducted in accordance with the International Organization for Standardization 14040/14044 standards using the OpenLCA software and the Ecoinvent database. Ten key HD products were analyzed across all life cycle stages from raw material extraction to disposal.

**Results:**

The dialyzer and bicarbonate cartridge were the largest contributors, together accounting for ∼70% of total carbon emissions. Additional impacts arose from saline solution, blood tubing, and dialysate bags. Plastic production, transport, and waste incineration were key drivers of environmental burden. Electricity use from HD machine operation also contributed substantially to total energy demand.

**Limitations:**

This study was based on data from a single center and relied on life cycle databases that may not fully capture variability in manufacturing and disposal processes. Some consumables and clinical variations were not included.

**Conclusions:**

HD procurement is a major source of environmental impact, largely driven by a small number of high-impact consumables. Targeted strategies including product redesign, reduction of single-use materials, and improved waste management may reduce the environmental footprint of dialysis care.

## Plain-Language Summary

Hemodialysis is a life-saving treatment for patients with kidney failure, but it also uses large amounts of water, energy, and single-use materials. This study examined the environmental impact of dialysis by analyzing the full life cycle of commonly used equipment and consumables, from production to disposal. We found that a small number of items, particularly the dialyzer and bicarbonate cartridge, contributed most of the environmental burden. Waste disposal and plastic use were also important factors. These findings show that changes in how dialysis products are designed, used, and disposed of could reduce environmental impact. Improving sustainability in dialysis is important for both patient care and reducing the wider impact of health care on the environment.

The impact of health care on global environmental sustainability has become a focal point for both research and policy reform, particularly in treatments associated with chronic conditions such as kidney failure. Hemodialysis (HD), a lifeline for patients with kidney failure, is notably resource intensive, requiring significant amounts of water and energy, and generating substantial medical waste. This treatment process not only demands a continuous supply of electricity to operate machinery but also consumes up to 500 L of water per session, contributing to a considerable environmental footprint.^1^

The ecological considerations of HD extend beyond the use of water and energy. The procurement and disposal of single-use medical consumables such as dialyzers, bloodlines, and other related materials play a significant role in environmental degradation. These components, essential for the dialysis process, are typically made from nonbiodegradable materials that contribute to the volume of medical waste.^2^ This waste not only requires specialized, costly disposal methods but also poses long-term environmental hazards.^3^

Current sustainability practices within the health care sector are under scrutiny, with a push toward reducing the carbon footprint and improving waste management strategies. In the context of HD, this involves a detailed examination of every stage of the medical supply chain, from the extraction and processing of raw materials used in medical products to their end-of-life disposal.^4^ Such an analysis is critical to identify key areas in which interventions can yield significant environmental benefits.^5^

This study employs a comprehensive life cycle assessment (LCA) to evaluate the environmental impacts of HD with a focus on its consumables and equipment. The LCA methodology adheres to the International Organization for Standardization 14040/14044 standards, which are internationally recognized frameworks that define the principles, system boundaries, and methodological requirements for conducting and reporting LCAs. These standards ensure transparency, consistency, and comparability across LCA studies, providing a detailed insight into the carbon emissions, water use, and waste production associated with HD.^6^ By mapping these impacts across the treatment’s life cycle, this study aimed to pinpoint significant contributors to environmental degradation and explore viable alternatives to current practice.

This study is part of the KitNewCare project, which seeks to reduce the environmental impact of kidney care through sustainable innovations.^7^ The focus in this study is on analyzing the procurement of HD consumables at the Nephrology Dialysis and Kidney Transplant Unit, University Hospital Policlinico of Modena, Modena, Italy. By evaluating the environmental footprint of each product used in HD, this study aimed to inform procurement practices that could significantly lower the environmental impact of dialysis treatment.^8^

## Methods

This study was conducted between April and July 2024 at the Nephrology Dialysis and Kidney Transplant Unit, University Hospital Policlinico of Modena, Modena, Italy, employing an LCA methodology in line with the International Organization for Standardization 14040/14044 standards,^9^ following an existing study in the KitNewCare project, which is looking at the environmental impact of the entire HD treatment. The analysis targeted the environmental impacts of procurement activities associated with HD, including the full life cycle of products from raw material extraction to end-of-life disposal.

### Procurement Data Collection

A detailed data set of all consumables used in HD was assembled, focusing on those with significant environmental impacts. Data sources included product records, supplier communications, and clinical staff input. Key elements included consumable quantities, material composition, manufacturing location, and transport logistics.

### Material Analysis and Product Breakdown

HD consumables were torn down (dismantled and analyzed) to identify material-specific contributions to environmental impacts. Products were disassembled to determine material composition, with components weighed and classified based on material type, including packaging.

### Life Cycle Impact Assessment

Environmental impacts were modeled for each HD product life cycle using the OpenLCA software (Greendelta).^10^ Processes were created for raw material extraction, manufacturing, transportation, use, and disposal, aligning with the study’s focus on procurement. Analysis was performed across multiple categories, including global warming potential, water use, and energy resources, using the Ecoinvent database version 3.11 (The Ecoinvent Association).^11^ The functional unit was defined as the complete set of consumables required for 1 HD patient over a 1-year treatment period.

Based on these findings, the following process was applied to select products for the detailed LCA in this study. Products were selected based on contribution to the overall carbon footprint and frequency of use, ensuring inclusion of both high-impact and commonly used consumables. This approach ensured that the LCA focused on the products with the greatest potential for environmental impact reduction through targeted optimization.

### Transportation Impact Assessment

Transportation-related emissions were analyzed to capture the environmental footprint of logistics. Transport distances from manufacturing facilities to Nephrology Dialysis and Kidney Transplant Unit, University Hospital Policlinico of Modena, Modena, Italy, were calculated using Google Maps and FluentCargo.com.^12^^,^^13^ Details on the modes of transport and associated fuel types were sourced from supplier data and literature.^14^ Transportation of medical waste to incineration or recycling facilities was also included.

### Waste Management Analysis

Disposal methods for HD-related waste were assessed to understand their environmental implications. Materials were classified into health care waste, general waste, and recyclables based on hospital waste management protocols. Environmental impacts of incineration, landfill, and recycling were modeled using local waste management data and Ecoinvent processes.

### Stakeholder Collaboration

Engagement with key stakeholders, including hospital administration staff, clinical staff, and waste management providers, ensured the accuracy and completeness of the data. Stakeholder input also supported the identification of optimization opportunities within the procurement pathway.

### System Boundary

Figure S1 illustrates the system boundaries defined for this study, encompassing all stages from raw material extraction to end-of-life disposal. This cradle-to-grave approach captures the full environmental burden of each product used in HD, including specific material types, manufacturing methods, transport distances, usage profiles, and waste treatment.

### Impact Categories

The following midpoint impact categories were evaluated^15^: global warming potential (kg CO_2_-equivalent [kg CO_2_-eq]); total greenhouse gas emissions, primary energy demand (MJ); total energy consumed during product life cycle, water use (m^3^); and water consumed during production and packaging.

### Ethics

The local ethics committee was consulted and this study was deemed not to require institutional review board approval.

Informed consent was not required as the study did not involve patient-level data or identifiable information.

### Functional Unit

The functional unit for this study was defined as the complete HD treatment for a single patient over a 1-year period. This allows for a standardized comparison of the environmental impact between different patient pathways.

## Results

### Product Overview

To improve clarity and generalizability, product names were anonymized into generic categories. Table 1 describes the nomenclature used throughout this section.

**Table 1:** Product Descriptions and Generic Names

Generic Name	Description
Dialyzer	Single-use, hollow-fiber polysulfone-derived high-flux dialyzer that removes solutes and excess water from the blood during a HD session
Bicarbonate cartridge (750 g)	Single-use cartridge of sodium bicarbonate powder used to prepare dialysate throughout a HD session
Saline solution (500 mL)	Sterile 0.9% sodium chloride bag for priming, rinsing lines, and managing hypotension during treatment
Blood tubing set	Disposable tubing assembly that connects the patient’s vascular access to the HD machine
Acid concentrate bag (4.7 L)	Single-use acid concentrate supplied in a flexible bag used to prepare dialysate throughout HD treatment
HD machine (build + operation)	Integrated HD unit; impact includes materials and manufacturing of the machine plus its electricity use
Sterile gauze	Cotton pads used for site cleaning, dressing, and minor blood absorption during and after dialysis
20-mL syringe	Single-use syringe with Luer-lock fitting for administering saline, anticoagulant, or medication during a session
Surgical drape	Sterile sheet providing a barrier around the access site, maintaining an aseptic field
Infusion set	Tubing and connector set for administering saline during dialysis

Abbreviation: HD, hemodialysis.

### Total Carbon Emissions and Contribution Analysis

Figure S2 and Table S1 present the total carbon footprint (kg CO_2_-eq) associated with the procurement and use of HD products over 1 year for a single patient. The dialyzer contributed the largest share (200.7 kg CO_2_-eq), closely followed by the bicarbonate cartridge (198.2 kg CO_2_-eq). Together, these 2 consumables accounted for nearly two-thirds of the total product-related emissions. The saline solution (500 mL) (47.4 kg CO_2_-eq), blood tubing set (43.5 kg CO_2_-eq), and acid concentrate bag (4.7 L) (36.0 kg CO_2_-eq) also contributed substantially. The HD machine contributed ∼0.47 kg CO_2_-eq/treatment, based on an estimated 24.4 kg CO_2_-eq/y from both manufacture and operation. Among smaller but frequently used items, the sterile gauze (23.7 kg CO_2_-eq), 20-mL syringe (22.9 kg CO_2_-eq), surgical drape (20.0 kg CO_2_-eq), and infusion set (19.0 kg CO_2_-eq) showed significant cumulative impacts owing to their high usage rates throughout the year.

The dialyzer showed the highest total plastic-related emissions (122.7 kg CO_2_-eq), driven by its large volume of synthetic polymer use. It also contributed significantly to waste emissions (71.0 kg CO_2_-eq), reflecting incineration of mixed plastic and biological material. In contrast, the bicarbonate cartridge’s emissions were dominated by nonplastic materials (140.6 kg CO_2_-eq), likely owing to chemical content and container production.

Among all components, the dialysate bag (4.7 L) was transport intensive (24.8 kg CO_2_-eq), attributable to its high volume and weight per treatment. Plastic and waste emissions were also notable for this item (13.8 kg CO_2_-eq and 25.4 kg CO_2_-eq, respectively), highlighting the environmental burden of single-use fluid packaging. The blood tubing set and 20-mL syringe also showed substantial emissions from both plastic and waste categories, highlighting their role in the environmental impact of consumables.

The HD machine’s total embodied emissions were relatively moderate compared with single-use consumables, contributing ∼24.4 kg CO_2_-eq/patient-year. These emissions arose from both the materials and processes involved in manufacturing the machine, as well as its end-of-life treatment. Specifically, emissions stemmed from plastic components (4.0 kg CO_2_-eq), steel, and other metals such as aluminum and copper used in the frame and internal components (27.1 kg CO_2_-eq), manufacturing and assembly processes (0.38 kg CO_2_-eq), and end-of-life waste management (4.0 kg CO_2_-eq).

The 4.0-kg CO_2_-eq emissions from end-of-life treatment arose from the incineration or landfill of nonrecyclable components (eg, plastic casing), energy use in dismantling, and limited recycling of metals. These values were derived from Ecoinvent database processes, modeling standard European waste management practices.

It is important to note that the machine’s embodied emissions are offset over its full expected life span. We assumed 5 years of use at a rate of 936 treatments/y (3 sessions daily, 6 days/wk), totaling 4,680 sessions. The portion attributed to 1 patient receiving 156 sessions/y was included in this analysis.

Although the per-session embodied emissions from materials and manufacture were low (∼0.16 kg CO_2_-eq), electricity use during operation remained the dominant contributor at 21.5 kg CO_2_-eq/patient-year. This highlights the importance of an energy-efficient HD machine design and the use of renewable electricity in dialysis units.

Smaller items such as the sterile gauze, infusion set, and surgical drape individually contributed to lower emissions but collectively added up through the high frequency of use. The surgical drape and infusion set both exceeded 7.5 kg CO_2_-eq from plastic alone.

### Energy Resource Use

Figure S3 and Table S2 display the contribution analysis for energy resources among the products. The dialyzer contributed the highest primary energy demand across all HD products (2,716 MJ), followed closely by the bicarbonate cartridge (2,259 MJ). These figures resulted primarily from the energy intensity of raw material extraction and plastic production. The HD machine ranked third overall (∼370 MJ), but nearly 90% of this was due to electricity consumed during operation (328.6 MJ annually/patient).

The dialysate bag (4.7 L) also required significant energy (578 MJ), driven by a combination of plastic manufacturing (107.5 MJ) and material inputs (401.4 MJ), with transport (5.6 MJ) and waste treatment (18.6 MJ) contributing modestly.

Smaller consumables such as the surgical drape (333 MJ), infusion set (200 MJ), and syringe (224 MJ) also presented meaningful energy burdens when used frequently. Energy use from manufacturing methods, although minor compared with material production and electricity, was notable for the bicarbonate cartridge (119.8 MJ) and blood tubing set (31.9 MJ), owing to their complexity and sterilization requirements.

### Water Use

Figure S4 and Table S3 present a contribution analysis of water use across the selected HD products, highlighting the components that were responsible for the highest water consumption over a 1-year treatment period. Water consumption was highest for the sterile gauze (∼324.9 m^3^) owing to the high water demand in cotton textile production and bleaching processes. This was followed by the bicarbonate cartridge (324.8 m^3^), in which sodium bicarbonate production was particularly water intensive.

The dialyzer also showed a significant water footprint (42.9 m^3^), driven largely by plastic processing and end-of-life treatment. The surgical drape (∼5.94 m^3^) and infusion set (∼5.03 m^3^) were notable for their complex plastic blends and nonwoven materials.

Despite its fluid content, the dialysate bag had a relatively low water footprint (**∼**2.17 m^3^) owing to more efficient production pathways and smaller packaging components per unit weight compared with cotton- or sodium-based products.

Water use from HD machine operation was modest (∼4.15 m^3^) and primarily reflected electricity consumption and component manufacturing. Smaller items such as syringes and blood tubing contributed under 3 m^3^ each, although when aggregated across sessions, their cumulative impact could be substantial.

## Discussion

In this LCA of HD procurement, we found that dialysis consumables accounted for 635 kg CO_2_-eq of greenhouse gas emissions, 8,128 MJ of primary energy demand, and 777 L of water use/patient-year. These findings highlight the substantial environmental footprint associated with dialysis consumable procurement.

This study reinforces the growing evidence that procurement activities are major contributors to environmental impact in health care, particularly within HD. HD equipment and consumables, together with care processes, need to be advanced to reduce waste generation, improve recyclability, and optimize water purification efficiency and water use.^16^ Our LCA of HD consumables and equipment in the Italian dialysis center showed that just 2 products, the bicarbonate cartridge and the dialyzer, contributed around 70% of procurement-related carbon emissions for 1 patient’s annual treatment. This reflects broader calls for reform in dialysis procurement to build climate-resilient nephrology services.^17^

### Product-Specific Contributions and Packaging

The bicarbonate cartridge’s environmental impact was closely tied to its material composition and transport intensity. The combination of plastic housings, sodium bicarbonate powder, and protective packaging contributed to a high resource burden. Similarly, dialyzers composed of polysulfone membranes and polyurethane housings involved complex multimaterial constructions that were difficult to recycle and energy intensive to manufacture.^18^ These materials, coupled with incineration at end of life, added significantly to the overall footprint.

The acid concentrate bag (4.7 L), although heavy and transport intensive as a single-use item, was found to be relatively efficient per liter of the dialysate produced. Each 4.7-L acid bag typically yielded around 150 L of dialysate when mixed with bicarbonate concentrate and osmotized water. However, as highlighted by Di Chiaro et al,^19^ a centralized acid distribution system can significantly reduce the environmental burden of acid delivery by eliminating single-use packaging and reducing transport weight. This suggests that central systems should be prioritized where feasible.

Although packaging impacts were not measured separately in this study, they remain a frequently overlooked contributor to environmental burden. Previous research has shown that, when aggregated across repeated treatments, packaging can account for hundreds of kg CO_2_-eq annually.^17^ A future analysis could isolate and compare the footprint of primary and secondary packaging associated with different delivery models.

### Waste Management and Material Impacts

End-of-life treatment, particularly incineration, significantly affects impact categories such as carbon emissions and water use. Polyvinyl chloride--containing items such as blood tubing and infusion sets are particularly problematic owing to dioxin emissions during incineration.^20^ Although polyvinyl chloride recycling is technically possible, uptake in clinical settings remains limited.^21^ The use of safer alternatives, such as thermoplastic elastomers or biodegradable polymers, may reduce these risks over time.^21^^,^^22^

Even smaller consumables such as syringes, gauze, and drapes contributed notably to environmental impacts owing to their material composition and frequent use. In most settings, including Italy, these items are classified as hazardous medical waste, regardless of actual infection risk, and are typically treated by autoclaving after shredding, followed by landfill or energy recovery.

Although some items may be perceived as low risk, definitive classification is difficult, and health care institutions must adhere to strict national regulations. The process of reclassifying certain waste streams, where clinically appropriate and legally permissible, has been proposed as a means of reducing carbon emissions and waste treatment burdens.^19^^,^^20^ Examples such as Australia’s pilot projects to recycle treated dialysis waste into concrete demonstrate the potential of alternative pathways, but widespread implementation remains dependent on regulatory adaptation and clinical assurance of safety.^23^^,^^24^

### Importance of a Full Impact Assessment

A strength of this study was its inclusion of multiple environmental indicators beyond carbon emissions, such as energy demand and water use. These are particularly relevant in dialysis owing to high material and energy input requirements. For instance, gauze and bicarbonate production processes are water intensive. Meanwhile, the HD machine consumed significant electricity over the course of a year, reinforcing the importance of including both build-phase and operation-phase impacts in LCAs.^25^^,^^26^

### Opportunities for Reductions

The results point to several strategies to reduce the environmental impact of HD, particularly by targeting the following 2 most carbon-intensive products: (1) the bicarbonate cartridge and (2) the dialyzer.

In clinical practice, it is common for a certain amount of bicarbonate powder to remain unused at the end of each dialysis session. This waste contributes unnecessarily to the carbon footprint and environmental toxicity, particularly when incinerated alongside its plastic cartridge. Although this study did not analyze residual bicarbonate disposal impacts directly, this is an area for further research. One solution is to tailor the bicarbonate dose to the precise requirement of each session to minimize leftover product.

Reusing dialyzers for the same patient has been proposed as a strategy to reduce environmental impacts by lowering plastic waste and manufacturing burden. However, reuse is banned in Italy and many other countries, owing to the risks of infection, structural degradation of membranes, and increased mortality reported in past studies.^27^ Moreover, dialyzer reprocessing requires additional chemicals, water, and energy for disinfection, which would introduce new environmental burdens, including CO_2_ emissions from sterilant. Therefore, although reuse may reduce material waste, its overall environmental and clinical tradeoffs must be carefully evaluated in any future analysis.^28^

In addition to product-specific strategies, broader system-level interventions could support environmental improvements. This is outlined in Table 2.^19^, ^20^, ^21^^,^^23^, ^24^, ^25^, ^26^^,^^29^^,^^30^

**Table 2:** Description of Interventions

Intervention	Description
Product redesign	Use of monomaterials and PVC-free alternatives to improve recyclability and reduce toxicity^21^
Localization	Sourcing or manufacturing closer to point of use to reduce transport emissions^29^
Energy transition	Decarbonization of electricity supply used in dialysis units^25^^,^^26^
Modular systems	Designing products for disassembly and material separation to enable recycling^30^
Optimized packaging formats	Use of bulk or optimized volumes to reduce packaging waste and transport burden^19^
Waste pathway reform	Development of safe pathways to reduce hazardous waste classification and enable material recovery^20^^,^^23^^,^^24^

Abbreviation: PVC, polyvinyl chloride.

### Comparison With Previous Studies

Our findings align with earlier HD LCAs conducted in the United Kingdom and Australia, which also identified dialyzers and dialysates as key hotspots.^5^^,^^31^ For example, Agar et al^5^ emphasized dialyzer disposal and water waste challenges in Australia, whereas Connor et al^31^ highlighted the carbon intensity of dialysate and travel in UK systems. This study contributes additional granularity by distinguishing product-level contributions and detailing energy and water use.

The high electricity demand of HD machines supports findings by Gauly et al,^16^ who argue for a smarter machine design and renewable energy use. Meanwhile, calls by Piccoli et al^17^ and Sousa et al^22^ to phase out polyvinyl chloride and improve waste treatment align closely with this study’s proposed actions.^26^

By identifying transport, plastic, and waste treatment as high-impact stages in the life cycle, this study provides a roadmap for product and system redesign in dialysis and supports broader sustainability goals in health care.

## Limitations

Although this study provides a comprehensive analysis of the environmental impacts associated with HD, there are several limitations that should be acknowledged. First, the procurement data collected were specific to the Nephrology Dialysis and Kidney Transplant Unit, University Hospital Policlinico of Modena, Modena, Italy, which may not fully represent global practices or the variability in HD products across different regions and health care systems. Results may vary significantly in centers using different materials, suppliers, or treatment technologies.

In conclusion, this comprehensive LCA of HD treatments highlights significant environmental burdens associated with key HD products. Products such as the dialyzer and bicarbonate cartridge (750 g) are major contributors to carbon emissions and resource depletion. The study highlights the urgent need for sustainable practices in HD, including the adoption of alternative materials with lower environmental impacts, improvements in resource use efficiency, and sustainable manufacturing processes. By implementing these changes, the HD sector can significantly reduce its environmental footprint, advancing global health care sustainability and aligning with broader environmental goals.
