# Supplementary material for: Procurement and Waste in Hemodialysis: A Detailed Life Cycle Assessment to Reduce Environmental Impact
Source: Kidney Med. 2026 Sep 7;8(11):101517. doi: 10.1016/j.xkme.2026.101517 (PMC13694722; doi:10.1016/j.xkme.2026.101517)
Supplement: Supplementary file 1 [file mmc1.pdf]

Supplementary Material

Larkin, J. (2024) 'Life Cycle Assessment Dataset For Haemodialysis in Modena'. Brett Duane. doi: 10.5281/zenodo.14259304.

Larkin, J. (2024) 'Product Images for Life Cycle Assessment Dataset For Peritoneal Dialysis in Modena'. Brett Duane. doi: 10.5281/zenodo.14258920

	Dialyser	Bicarbonate cartridge (750 g)	Saline solution (500 ml)	Blood tubing set	Dialysate bag (4.7 L)	HD machine (build + operation)	Sterile gauze	20 ml syringe	Surgical drape	Infusion set
transport, freight	6.38	26.19	24.78	2.09	0.40	0.06	0.07	1.28	0.20	0.20
other materials	-	140.62	0.24	0.01	27.12	2.57	17.48	1.23	7.81	0.99
plastic	122.69	23.24	13.81	13.77	4.05	0.10	1.53	7.39	3.73	7.50
waste	71.03	0.00	7.92	25.40	4.01	0.14	3.33	10.23	7.72	9.43
manufacturing method	0.61	8.16	0.61	2.24	0.38	0.08	1.30	2.17	0.75	0.84
electricity	-	-	-	-	-	21.54	-	-	-	-

Table S1 Contribution Analysis (Carbon Emissions)

Lifecycle Stage	Dialyser	Bicarbonate cartridge (750 g)	Saline solution (500 ml)	Blood tubing set	Dialysate bag (4.7 L)	HD machine (build + operation)	Sterile gauze	20 ml syringe	Surgical drape	Infusion set
transport, freight	89.60	367.80	336.80	29.40	5.60	0.73	0.90	17.90	0.20	2.80
other materials	2,247.60	1,589.90	242.90	87.30	401.40	36.10	173.30	83.50	187.60	60.40
plastic	122.70	181.80	160.40	102.50	107.50	-	38.60	40.70	93.70	79.50
waste	347.30	-	36.70	124.20	18.60	0.63	15.40	50.00	37.80	46.10
manufacturing method	8.90	119.80	7.60	31.90	4.80	0.22	2.40	31.90	13.90	12.00
electricity	-	-	-	-	-	328.60	-	-	-	-

Table S2 Contribution Analysis (Energy Resources)

Lifecycle Stage	Dialyser	Bicarbonate cartridge (750 g)	Saline solution (500 ml)	Blood tubing set	Dialysate bag (4.7 L)	HD machine (build + operation)	Sterile gauze	20 ml syringe	Surgical drape	Infusion set
transport	2.95	2.06	1.68	0.16	0.03	0.00	0.01	0.10	-	0.02
plastic	34.81	6.12	7.74	4.48	2.17	-	0.80	2.25	3.54	2.49
materials	-	314.28	0.82	-	46.92	0.98	322.51	-	-	1.38
waste	5.04	1.37e-06	0.53	1.80	0.27	0.01	0.22	0.73	0.55	0.67
manufacturing	0.15	1.96	0.16	0.55	0.10	0.00	-	0.52	1.86	0.21
electricity	-	-	-	-	-	3.17	-	-	-	-

Table S3 Contribution Analysis (Water Use)

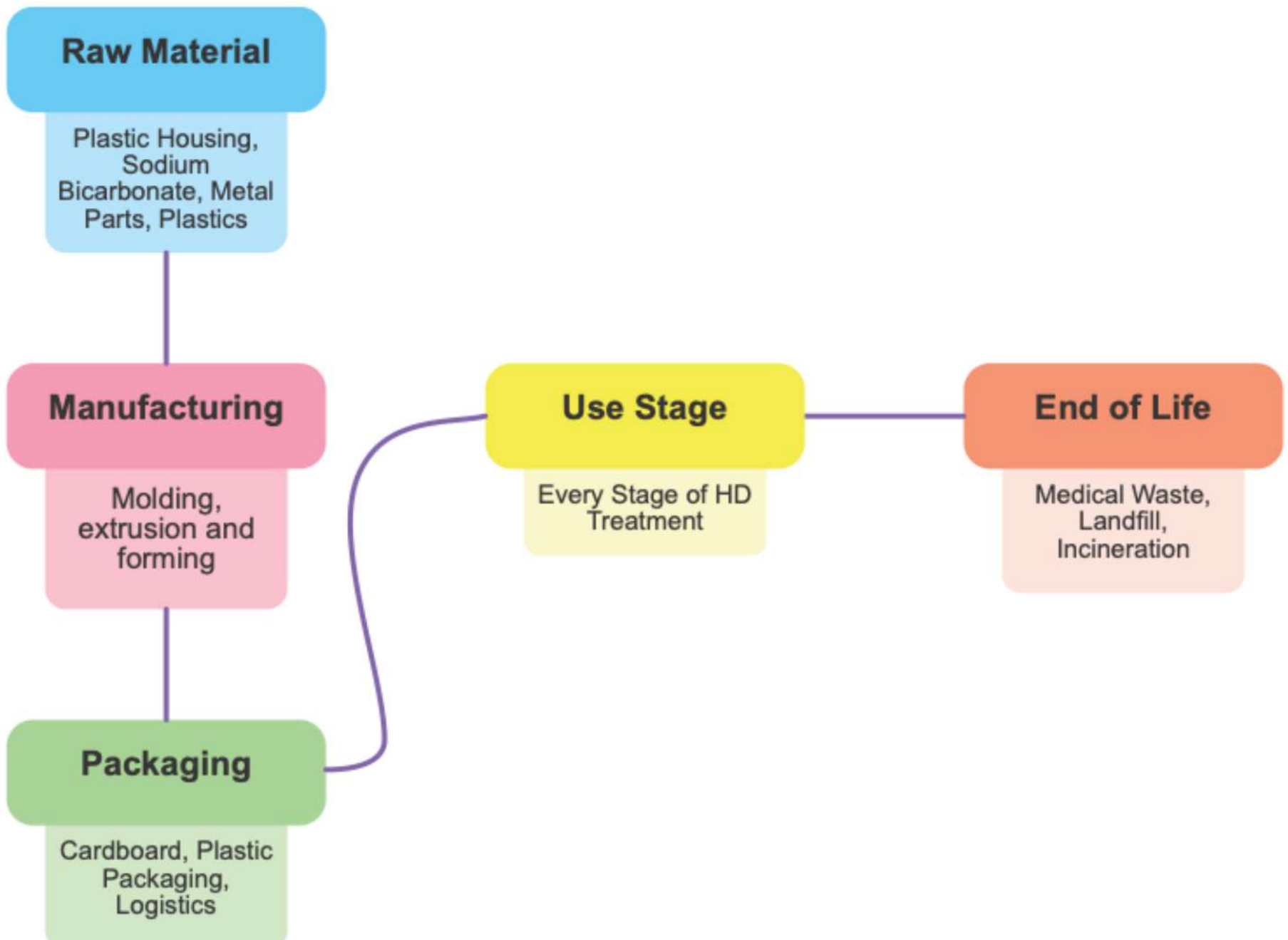

Figure S1 System Boundary

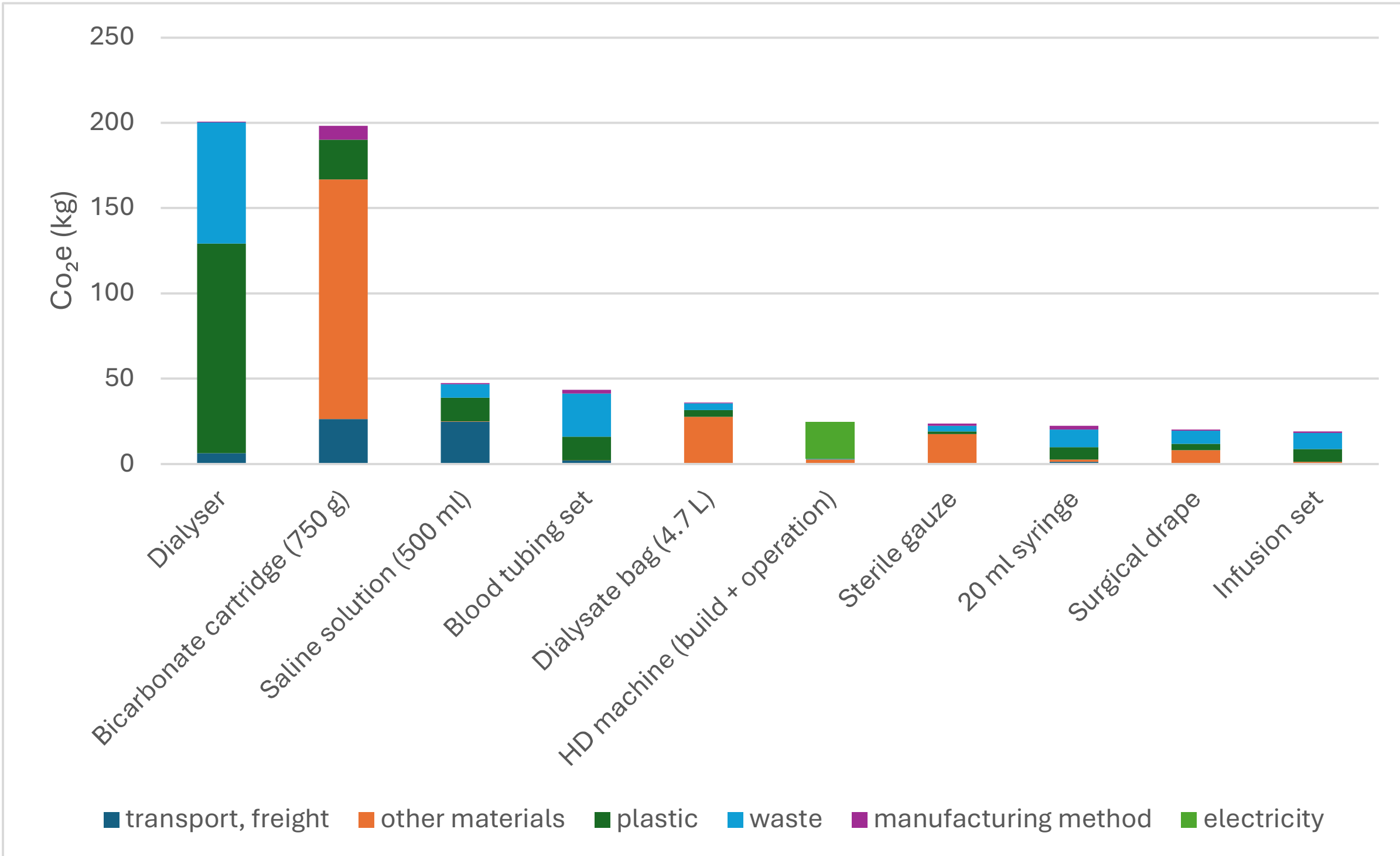

Figure S2 Contribution Analysis (Carbon Emissions)

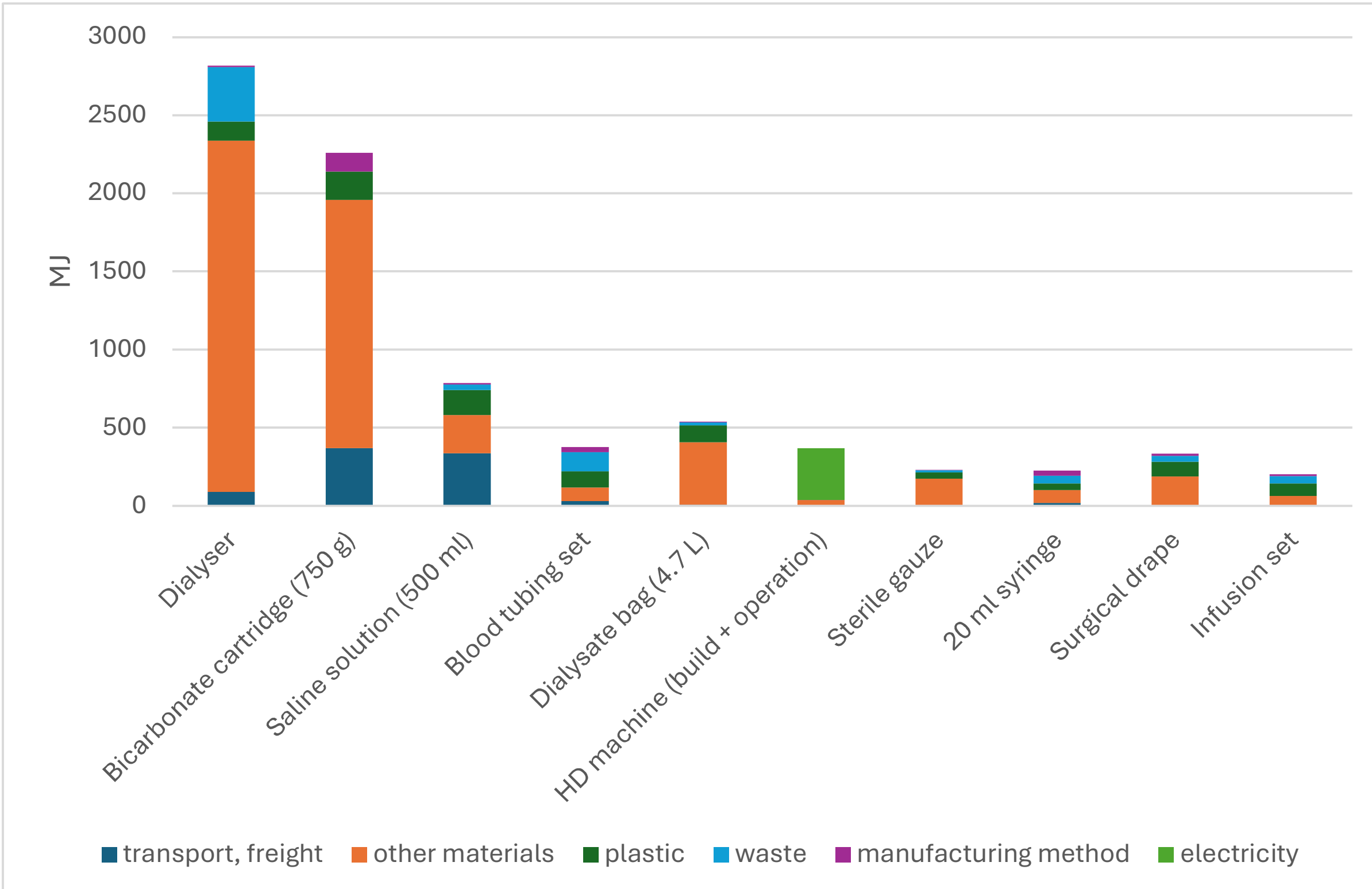

Figure S3 Contribution Analysis (Energy Resources)

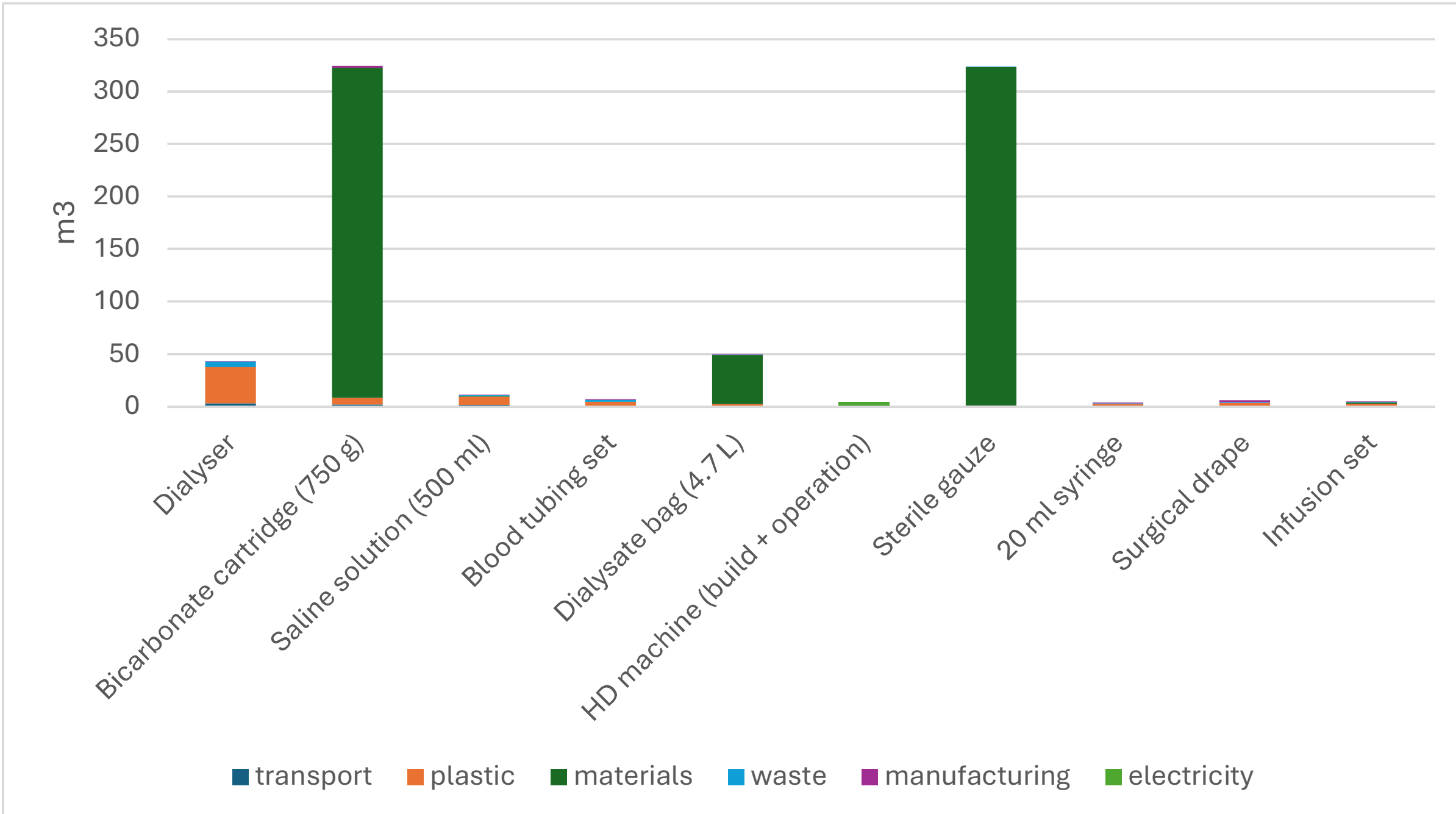

Figure S4 Contribution Analysis (Water Use)
